# Modulating Crystallization and Defect Passivation by Butyrolactone Molecule for Perovskite Solar Cells

**DOI:** 10.3390/molecules28145542

**Published:** 2023-07-20

**Authors:** Fengyou Wang, Jinyue Du, Chenyu Zhao, Yutao Li, Maobin Wei, Huilian Liu, Jinghai Yang, Lili Yang

**Affiliations:** 1 Key Laboratory of Functional Materials Physics and Chemistry of the Ministry of Education, Jilin Normal University, Changchun 130103, China; wfy@jlnu.edu.cn (F.W.); jydu_jlnu@163.com (J.D.); jlsdzccw@126.com (M.W.); 2 National Demonstration Center for Experimental Physics Education, Jilin Normal University, Siping 136000, China

**Keywords:** polycrystalline thin film, surface passivation, nucleation, nonradiative recombination, perovskite solar cells

## Abstract

The attainment of a well-crystallized photo-absorbing layer with minimal defects is crucial for achieving high photovoltaic performance in polycrystalline solar cells. However, in the case of perovskite solar cells (PSCs), precise control over crystallization and elemental distribution through solution processing remains a challenge. In this study, we propose the use of a multifunctional molecule, α-amino-γ-butyrolactone (ABL), as a modulator to simultaneously enhance crystallization and passivate defects, thereby improving film quality and deactivating nonradiative recombination centers in the perovskite absorber. The Lewis base groups present in ABL facilitate nucleation, leading to enhanced crystallinity, while also retarding crystallization. Additionally, ABL effectively passivates Pb^2+^ dangling bonds, which are major deep-level defects in perovskite films. This passivation process reduces recombination losses, promotes carrier transfer and extraction, and further improves efficiency. Consequently, the PSCs incorporating the ABL additive exhibit an increase in conversion efficiency from 18.30% to 20.36%, along with improved long-term environmental stability. We believe that this research will contribute to the design of additive molecular structures and the engineering of components in perovskite precursor colloids.

## 1. Introduction

Organic metal-halide perovskites are becoming significant photovoltaic absorbers that have the potential to prepare high-efficiency solar cells at an affordable cost [1,2,3,4,5]. Due to their appealing optoelectronic characteristics, significant advancements have been made in recent years regarding the power conversion efficiencies (PCE) of perovskite solar cells (PSCs), which now boast record values exceeding 25% [6]. However, the low ionic migration energies along with the “soft” nature of the MHP also lead to unacceptable ion redistribution as well as phase separation in external stimulation [7,8]. Studies show that twelve intrinsic point defects will be generated in the MAPbI_3_ grains, such as interstitials (interstitial MA, interstitial Pb, and interstitial I), vacancies (MA-vacancy, Pb-vacancy, and I-vacancy), and substitution (MA substitute Pb, Pb substitute MA, MA substitute I, Pb substitute I, I substitute MA, and I substitute Pb) [9]. The dangling bonds located at the grain surfaces, involving lead clusters, uncoordinated Pb^2+^, negative unbonded halide ions, and organic-cation vacancies, can also contribute to the nonradiative recombination and the annihilation of charges [5]. These recombinations will trigger the open-circuit voltage loss of the device, playing as an obstacle toward the theoretical efficiency limit of 30%. In addition, the defects within the perovskite layer are also one main source of the instability of the device due to the defects-induced perovskite-framework collapses [10]. Therefore, it is critical to terminate these different kinds of defects simultaneously to reduce the charge recombinations and reinforce the framework of the perovskite, thereby wholly enhancing the efficiency of the PSCs.

To achieve this goal, more strategies, including additive engineering, dimensionality modulation, and post-treatment, have been made to realize crystallization modulation, surface passivation, device self-sealing, etc., aiming to control the native defect density during the perovskite growth and eliminate the surface dangling bonds on the film surface [11,12,13,14,15,16]. Among them, additive engineering is mostly used because it can trigger different effects on perovskite films, from nucleation to the final ripening process [17,18,19]. For example, several inert large molecules, supramolecules, and polymers have been explored to target the undesired defects by adding them to the perovskite precursor solutions [1,20,21,22,23]. The defects at the grain boundaries can be finely passivated by forming hydrogen bonds or coordination bonds with the additive, and a moisture barrier can be built to improve the stability. Because the point defects at the bulk of the grains can rarely be terminated due to their large size, the additive is hardly incorporated into the perovskite lattice. Therefore, to further eliminate the defects induced by the solution-processed crystallization, halide acids, zwitterionic sulfamic acid, and citric acid have been used to promote the dissolution of precursor colloids, thus yielding the film with enhanced crystallization and fewer point defects [24,25,26,27,28]. However, the residual H_2_O in these acids is difficult to thoroughly remove from the perovskite precursor, and the grain boundaries cannot be self-sealed compared to the large molecular additives, which degrades the perovskite film stability. Hence, finding a suitable additive that can enable good crystallization and passivate the defects at the grain boundaries, along with building a moisture barrier, is a meaningful route to enhance the perovskite film stability. 

Herein, we successfully screened a lactone hydrobromide additive, termed α-amino-γ-butyrolactone (ABL), containing lactones and -NH_2,_ to improve the quality and stability of the perovskite films. In contrast to existing large-size molecular additives and inorganic salt additives, this study proposes the use of small molecular additives to treat perovskite precursors. ABL, as one type of small molecule, has minimal steric hindrance. Moreover, through ligand engineering, they can exhibit excellent passivation capabilities, thereby enhancing the quality of perovskite films. The ABL with γ-butyrolactone can increase the solubility of perovskite intermediate in the solvent [29], decreasing the large colloidal content and resulting in better film morphology with increased grain size. Moreover, the Pb^2+^ dangling-bond defects at the grain boundaries can be terminated by the -NH_2_ and C=O groups of ABL, which reduces the nonradiative recombination loss. And the lactone of ABL with hydrophobic nature can serve as a moisture barrier at the grain boundaries and resist moisture invasion, which improves the stability of the films. The PCE of the planar MAPbI_3_ solar cells prepared with the ABL improved from 18.30% to 20.36%. Additionally, the modified solar cells showed improved environmental and thermal stabilities. 

## 2. Results and Discussion

**Figure 1:**
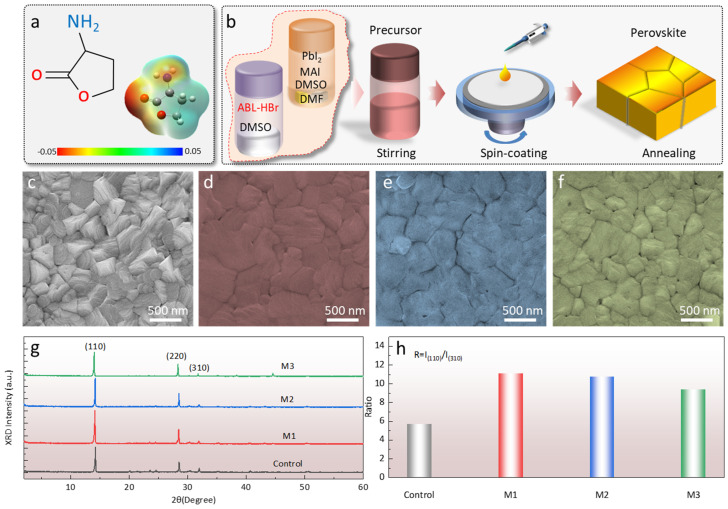
(**a**) Surface potential and structure of the ABL. (**b**) Schematic diagram of the fabrication processes of perovskite films with ABL additives. The SEM images of the perovskite films with different concentrations of ABL: (**c**) control, 0 mg/mL; (**d**) M1, 0.1 mg/mL; (**e**) M2, 0.5 mg/mL; and (**f**) M3, 1 mg/mL. (**g**) XRD patterns of the perovskite film with different concentrations of ABL. (**h**) The *R* values of the perovskite films with various concentrations of ABL additives.

The ABL is one kind of organic molecule that can be dissolved in the DMSO solvent. The chemical structure and simulated electrostatic potential (ESP) show that the negative potential is loading at the -NH_2_ and C=O, suggesting electron-donating characteristics. We blended the ABL with perovskite (MAPbI_3_) precursor and fabricated the perovskite by adopting a spin-coating approach (Figure 1b). The thin-film morphology is one of the critical factors affecting device performance. We optimized the perovskite morphology by adding different contents of ABL in the precursor, which were 0.1 mg/mL, 0.5 mg/mL, and 1 mg/mL, and the corresponding thin films were termed M1, M2, and M3, respectively. As shown in Figure 1c–f and Figure S1, the scanning electron microscope (SEM) demonstrated the grain size was remarkably enlarged when the ABL concentration was 0.1 mg/mL (M1), and the surface was much smoother than the control film. Further increasing the ABL concentration to 0.5 mg/mL and 1 mg/mL (M3) gradually yielded some small grains on the surface. This phenomenon can also be quantitatively verified by the atomic-force-microscope (AFM) images (Figure S2), which show that the root-mean-square (RMS) roughness declined from 17.47 (control) to 12.45 nm (M1) and then increased to 32.40 nm and 35.31 nm for M2 and M3, respectively. The increased RMS for M2 and M3 may have originated from the addition of excess ABL breaking the ordered sol–gel precursor phase, yielding an inhomogeneous distribution of perovskite and a smaller average grain size [28]. X-ray diffraction (XRD) was further performed to unveil the phase structure of the perovskite films (Figure 1g). The peaks at 14.2°, 28.5°, and 31.8° were indexed as the (110), (220), and (310) planes of the MAPbI_3_ film, respectively [30]. The M1 peak intensity was apparently 1.3 times higher than that of the control film, and the intensity gradually decreased with further increasing of the ABL content. Furthermore, the relative intensity of (110)/(310) in Figure 1h shows the grains of M1 had a preferred (110) orientation, indicating that the grain prefers to grow perpendicularly toward the substrate [31]. The UV-vis absorption spectra (Figure S3) exhibit that the absorption intensity for M1 was slightly higher than that of the control films, which may have originated from the improved crystallization. 

**Figure 2:**
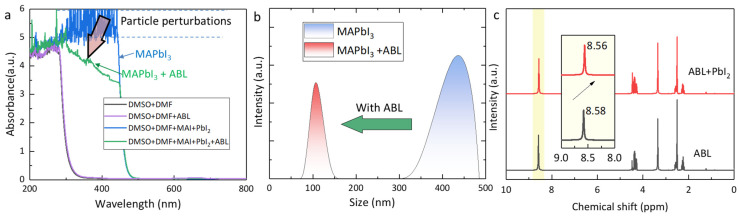
(**a**) UV–vis absorption spectra of the different solutions, including DMF + DMSO, DMF + DMSO + ABL, DMF + DMSO + MAI + PbI_2_, and DMF + DMSO + MAI + PbI_2_ + ABL. (**b**) DLS spectra of the MAPbI_3_ and MAPbI_3_–ABL solutions. (**c**) ^1^H NMR of ABL and ABL + PbI_2_. Inset: The scale magnification between 8 and 9 ppm.

We further investigated the precursor properties to figure out the reason for the better crystallinity of the MAPbI_3_ + ABL film. Actually, when we fabricated the perovskite precursor by dissolving MAI and PbI_2_ in the given solvent (DMF and DMSO), we produced a colloidal system instead of the real solution [32]. The perovskite nucleates from the colloidal cluster and the cluster distribution in the precursor should be carefully controlled to obtain uniform films. The γ-butyrolactone groups of the ABL endow ABL with the capability to dissolve the MAI and PbI_2_, which increases the precursor colloidal solubility and decreases the content of large colloids [33]. This can be visually presented by the changes in the UV-vis absorption of the precursors (Figure 2a). The UV-vis absorption spectra demonstrate colloid-sized perturbations, along with the absorbance intensity, at the wavelength range of 200–450 nm, which decreased after introducing ABL, suggesting a contracted colloidal cluster. Also, the dynamic-light-scattering (DLS) spectra of the two precursors (Figure 2b) show that the colloidal size of the MAPbI_3_ precursor with ABL (~170 nm) was smaller than that of the pristine precursor (~440 nm), which is consistent with the above results. In general, smaller colloids tend to require higher temperatures and longer durations for complete crystallization, leading to improved morphology by increased grain size and crystallinity [34]. Meanwhile, the small colloids can also trigger homogeneous nucleation for the perovskite precursor, improving the uniformity of the crystallization and elemental distribution [35,36]. 

Except for the nucleation process, we also collected the liquid ^1^H nuclear-magnetic-resonance (NMR) spectra of the ABL and the ABL + PbI_2_ solution in deuterated dimethyl sulfoxide-d_6_ (DMSO-d_6_) to investigate the scheme of the enlarged grain size for the ripened films (Figure 2c). With the PbI_2_ additive, the peak located at 8.58 ppm exhibits a downshift. This phenomenon can be ascribed to the interaction between the -NH_2_ and the Pb^2+^ in the perovskite precursor, which will delay the crystallization process of perovskite and is also beneficial to upgrade the crystallization of the films [33]. Therefore, the above research indicates that ABL can improve the quality of perovskite thin films by shrinking the colloidal particles in the precursor and prolonging the crystallization process.

**Figure 3:**
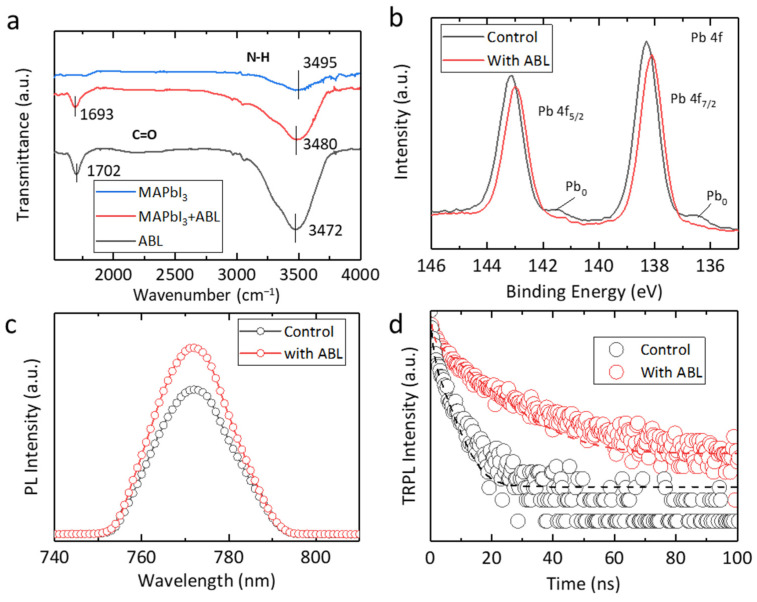
(**a**) The FTIR of the ABL, perovskite, and perovskite–ABL films. (**b**) The XPS of the perovskite films with and without ABL additives. (**c**) The room temperature PL spectra of the perovskite films with and without ABL additives. (**d**) The TRPL decay curves of the control film and the perovskite–ABL film.

The surface passivation effects for perovskite film brought by the ABL additive have also been studied. The Fourier transform infrared spectroscopy (FTIR) of the ABL and the perovskite films with and without ABL additive is shown in Figure 3a. The C=O stretching mode can be observed in both the ABL and MAPbI_3_ + ABL samples. Compared with the ABL, the C=O stretching mode of the MAPbI_3_ + ABL sample was downshifted from 1702 to 1693 cm^−1^, which refers to the interaction between the C=O of the ABL and the Pb^2+^ of the perovskite. In addition, the N-H stretching vibration mode for ABL, MAPbI_3_ + ABL, and MAPbI_3_ films are 3472, 3480, and 3495 cm^−1^, respectively. The wavenumber difference between MAPbI_3_ and MAPbI_3_ + ABL indicates the -NH_2_ group of the ABL can also terminate the Pb^2+^ of the MAPbI_3_, thus synergistically passivating deep-level defects within the film. The X-ray photoelectron spectroscopy (XPS) was also taken to elucidate the passivation details (Figure 3b). The XPS peaks of Pb 4f core singles of the MAPbI_3_ + ABL shift towards the lower binding energies. This is due to the passivation group of the ABL (C=O and -NH_2_) donating electrons to the Pb^2+^ defects of the MAPbI_3_, thus increasing the Pb 4f electron cloud density. Moreover, for the pristine MAPbI_3_, the Pb_0_ peaks at 141.4 and 136.6 eV can be observed, which is one type of defect caused by the evaporation of organic cations and halide anions [32,37]. While the Pb_0_ is apparently reduced for the MAPbI_3_ + ABL film, which suggests that the ABL cannot only terminate the Pb^2+^ dangling bonds but enhance the framework and restrain the defect generation. We then studied the perovskite film’s defect density by the space-charge–limited-current (SCLC) approach with an architecture of an FTO/SnO_2_/perovskite/PCBM/Ag device (Figure S4). The trap density declined from 2.73 × 10^16^ to 5.27 × 10^15^ cm^−3^ after introducing the ABL, indicating an effective passivation effect of the ABL. The steady-state photoluminescence (PL) and time-resolved PL (TRPL) are taken to reveal the carrier kinetics of the perovskite films (Figure 3c,d, respectively). The MAPbI_3_ + ABL film exhibits a higher PL intensity than that of the control film, implying that ABL, as an effective passivator, indeed suppresses carrier recombination by coordinating with the perovskite film, which is in agreement with the FTIR and XPS results. As for the TRPL, a bi-exponential model is adopted to fit the experimental decay curves. Table S1 summarizes the output parameters. The average decay time of the MAPbI_3_ + ABL films was 468.9 ns, longer than that of the pristine MAPbI_3_ film (152.7 ns), demonstrating a declined nonradiative recombination.

**Figure 4:**
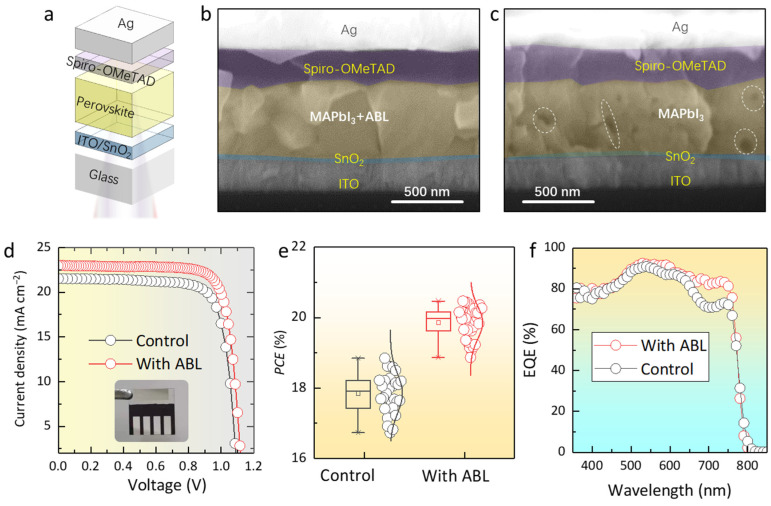
(**a**) Schematic diagram of the device structure. (**b**) The schematic diagram of the PSCs. The cross-sectional SEM images of the (**b**) perovskite–ABL device and (**c**) pure-perovskite device. The dotted circles highlights the pinhole within the perovskite films. (**d**) The illuminated *J-V* curves of the control and perovskite–ABL devices. Inset: the photograph of the device. (**e**) *PCE* distributions collected from 30 control and 30 ABL-based devices. (**f**) The *EQE* spectra of the PSCs with and without ABL additives.

With this information in hand, we then fabricated the planar PSCs with a configuration of ITO glass/SnO_2_/with or without ABL-modified MAPbI_3_/Spiro-OMeTAD/Ag (Figure 4a) to assess the impact of the ABL additive on the photovoltaic performance of the PSCs. Figure 4b,c show the cross-sectional SEM images of the PSCs with and without ABL. In agreement with the analysis above, the MAPbI_3_ + ABL layer shows better crystallization with denser and larger grains compared to the pristine perovskite layer, which is beneficial to improve the photogenerated charge transporting and suppress the recombination. The current-density–voltage (*J-V*) curves of the PSCs with and without ABL modification are exhibited in Figure 4d, and the detailed device output parameters are tabulated in Table S2. The control device yielded an efficiency of 18.30% (forward scan: *V_oc_* = 1.08V, *FF* = 75.37%, *J_sc_* = 21.32 mA cm^−2^, and *PCE* = 17.35%; reverse scan: *V_oc_* = 1.09 V, *FF* = 77.52%, *J_sc_* = 21.66 mA cm^−2^, and *PCE* = 18.30%). For the ABL-modified PSCs, the device possesses an apparently improved optimum *PCE* of 20.87% (forward scan: *V_oc_* = 1.12 V, *FF* = 79.06%, *J_sc_* = 22.99 mA cm^−2^, and *PCE* = 20.36%; reverse scan: *V_oc_* = 1.13 V, *FF* = 80.04%, *J_sc_* = 23.08 mA cm^−2^, and *PCE* = 20.87%). The enhanced *FF* and *V_oc_* are ascribed to the decrease of the defect density and nonradiative recombination, which originated from the passivation effect of the ABL. The improved *J_sc_* is correlated with the better crystallization of the perovskite–ABL layer with fewer voids, accelerating the charge transporting within the device. Meanwhile, to evaluate the reproducibility of the process, 60 devices (30 control PSCs and 30 ABL-based devices) were prepared, and the *PCE* statistics are demonstrated in Figure 4e. Compared with the control PSCs, the ABL-based devices achieved higher PCE values and narrower PCE distributions, validating good process parallelism. Moreover, the external-quantum-efficiency (EQE) spectra show that the quantum conversion efficiency of the device with ABL modification was notably improved in the range of 650–750 nm (Figure 4f), and the difference of the integrated photocurrent density is consistent with the *J-V* results (Figure S5). The better EQE performance is due to the MAPbI_3_ + ABL film being denser than the control film, which is beneficial to the extraction of charge at the rear side of the PSCs. 

**Figure 5:**
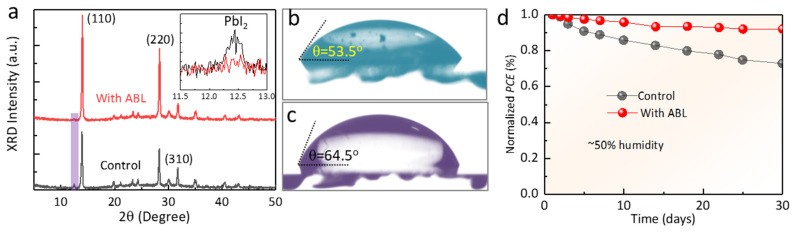
(**a**) The XRD patterns of the perovskite film with and without ABL after aging 30 days in ambient air condition. The water contact angles of the perovskite (**b**) with and (**c**) without ABL additives. (**d**) The normalized *PCEs* of the control device and the ABL-based device.

Except for the *PCE*, the stability of the PSCs is also a critical point to weigh the application potential [38,39,40]. We stored the bare perovskite films with and without ABL in the dark under a humidity of ~30% for 30 days (temperature ~25 °C). Figure 5a exhibits the corresponding XRD of the aged films. The control film exhibits a small PbI_2_ diffraction peak at ~12.5°, accounting for the decomposition of the perovskite by moisture erosion. In contrast, the ABL-modified films have no PbI_2_ diffraction peak. Meanwhile, we measured the water contact angles of the MAPbI_3_ and MAPbI_3_ + ABL films (Figure 5b,c). The angles increased from 53.5° (control) to 64.5° (with ABL) due to the inherent hydrophobic nature of the lactone. Therefore, these results indicate that ABL can enhance the stability of the perovskite film by enhancing crystallinity, passivating defects, and improving hydrophobicity. The photovoltaic performance of the unencapsulated PSCs was also examined at 25 °C in air with a humid environment of 50%. The normalized *PCE* trend curve shown in Figure 5d demonstrates that the *PCE* of the control device continued to decline to 72.8% of the initial efficiency. However, the PSCs with ABL modification retained more than 92.0% of the initial efficiency, exhibiting apparently improved stability. The enhancement of the stability is due to the interaction between ABL and perovskite suppressing the defect and strengthening the perovskite framework, as well as the increased hydrophobicity of the perovskite film preventing humidity-induced decomposition. 

## 3. Materials and Methods

Materials: Indium tin oxide (ITO) glass substrates, lead iodide (PbI_2_), methylammonium iodide (CH_3_NH_3_I, MAI), 4-*tert*-butypyridine and lithium bis (trifluoromethanesulfonyl) imide (Li-TFSI), SnO_2_ aqueous colloidal dispersion (15 wt%, ALFA), 2,2′,7,7′-tetrakis (*N*,*N*-dip-methoxyphenylamine) 9,9′-Spirobifluorene (Spiro-OMeTAD), and all anhydrous solvents were purchased from YOUXUAN Technology Co., Ltd. (Yingkou, China). The 2-amino-acetamidobenzotrifluoride (BDP) was purchased from TCI Shanghai. All chemicals and reagents were used as received from chemical companies without any further purification.

Fabrication of perovskite solar cells: SnO_2_ was used as ETL, which was coated on the ITO glass sheet by spin coating. The deposited ITO glass was transferred to a nitrogen-filled glove box (H_2_O and O_2_ < 1 ppm) to spin coat the perovskite film, which was prepared using 693 mg of PbI_2_, 245 mg of MAI, and different concentrations of ABL dissolved in a mixed solvent of dimethyl sulfoxide (DMSO) and dimethyl formamide (DMF) (*v*:*v* = 3:7). The 80 μL of the MAPbI_3_ precursor solution was deposited onto the ITO/SnO_2_ layer by a spin-coating process, i.e., 5500 rpm for 40 s, and 450 μL of chlorobenzene (CB) was poured on the rotating substrate 12 s after the start of the procedure. Subsequently, the obtained films were dried at 60 °C for 5 min and at 100 °C for 10 min. An amount of 75 μL of the hole-transport-layer solution; prepared by mixing 72.3 mg of Spiro-OMeTAD, 18.5 μL with a solution of 500 mg/mL Li-TFSI in acetonitrile, and 28.5 μL of 4-tertbutylpyridine in 1 mL of CB; was spin coated on the prepared MAPbI_3_ films at 3000 rpm for 30 s. Finally, 100 nm of Ag was thermally deposited under vacuum condition.

## 4. Conclusions

In summary, a multifunctional passivator of ABL was successfully proposed and introduced to the perovskite precursor to affect the crystallization process and passivate the defects. The Lewis base groups (-C=O, -NH_2_) of ABL can interact with the PbI_2_ in the precursor solution, thereby retarding the growth process and enhancing the crystallinity of the perovskite film. Moreover, the ABL can also passivate the Pb^2+^ dangling bonds, reducing the recombination losses and promoting carrier transfer and extraction. As an extra benefit, the inherent hydrophobicity of lactone endows the perovskite film with a moisture-resistant barrier, which promotes the humidity tolerance of the PSCs. The synergetic effect of the ABL improved the PCE of the MAPbI_3_-based solar cells from 18.30% to 20.36% with excellent environmental and thermal stabilities. We believe that this exploration can trigger more ideas for designing multifunctional molecules and improving the PCE and operational stability of PSCs in the future.

## Data Availability

The data presented in this study are available on request from the corresponding author. The data are not publicly available due to privacy.

## Supplementary Materials

The following supporting information can be downloaded at https://www.mdpi.com/article/10.3390/molecules28145542/s1, Figure S1: The grain sizes histogram for the perovskite film with different concentrations ABL additives; Figure S2: The AFM images of the perovskite films with different ABL additives: (a) control, 0 mg/mL, (b) M1, 0.1 mg/mL, (c) M2, 0.5 mg/mL, (d) M3, 1mg/mL; Figure S3: The absorption of the perovskite films with different concentrations ABL additives; Figure S4: The J–V curves of electronic-dominated devices with a structure of glass/ITO/SnO_2_/MAPbI_3_ (with- and without- ABL)/PCBM/Ag. The trap density can be determined from V_TFL_ by referring to the relationship V_TFL_ = eNtL^2^/2εε_0_, where the relative dielectric constant ε of MAPbI_3_, the ε_0_ is vacuum dielectric constant, and L is the thickness of the perovskite film; Figure S5: The integrated current density of the control and with ABL devices. Table S1: TRPL lifetimes of the MAPbI_3_ and MAPbI3 + ABL films; Table S2: The illuminated output paramenters of the PSCs with and without ABL.
