# Supplementary material for: Modulating Crystallization and Defect Passivation by Butyrolactone Molecule for Perovskite Solar Cells
Source: Molecules. 2023 Jul 20;28(14):5542. doi: 10.3390/molecules28145542 (PMC10383146; doi:10.3390/molecules28145542)
Supplement: Supplementary file 1 [file molecules-28-05542-s001.zip › molecules-2475241-supplementary.pdf]

Modulating crystallization and defect passivation by butyro-lactone molecule for perovskite solar cells

Fengyou Wang[1, 2], Jinyue Du[1], Chenyu Zhao[1], Yutao Li[1], Maobin Wei[1, 2], Huilian Liu[1, 2]*, Jinghai Yang[1, 2]*, Lili Yang[1, 2]*

1. Key Laboratory of Functional Materials Physics and Chemistry of the Ministry of Education, Jilin Normal University, Changchun 130103, China

2. National Demonstration Center for Experimental Physics Education, Jilin Normal University, Siping 136000, China

Corresponding author:

Huilian Liu, e-mail address: *lhl541@126.com*

Jinghai Yang, e-mail address: *jhyang1@jlnu.edu.cn*

Lili Yang, e-mail address: *llyang1980@126.com*

Supplementary note 1

Characterization: The scanning electron microscope (SEM) images and energy dispersive spectroscopy (EDS) linear-scan were taken using a Hitachi S-4800. NMR was measured by Bruker Avance III 400MHz, American. HRTEM images were measured by JEOL 2100F, Japan. AFM and KPFM were measured by Park NX20, Korea. The optical properties of the films were analyzed using an UV-Vis-near-infrared (NIR) spectrophotometer (Shimadzu, UV-3600Plus, Japan). Fourier transform infrared spectroscopy (FTIR) was measured by Nicolet iS10, America. Electron-only devices (ITO/SnO_2/perovskites/PCBM/Ag) were fabricated to calculate the trap state density of the devices. The defect density was determined by the equation for the trap-filled limit voltage. The crystal structure of the $MAPbI_3$ and $MAPbI_3$-BDP films was carried out by X-ray diffraction (XRD) (Japan Rigaku D/max-ga X-ray diffractometer) using Cu Kα (λ = 0.15406 nm) source. PL spectra were obtained using a PL microscopic spectrometer (FLS1000, China) with a 385 nm CW laser excitation source. The TRPL (FLS1000, China) were measured by using an excitation wavelength of 385nm. The main corresponding setup consisted of perovskite, mica-flakes. X-ray photoelectron spectroscopy. (XPS) was used to obtain the information on the chemical states of the control and $MAPbI_3$-BDP films (measured by Escalab250Xi, Germany). *J-V* characterizations was carried out was carried out under AM 1.5 G simulated sunlight illuminations (100 mW cm^{-2}, Model 94043A, Oriel, American). The spectral responses were obtained from an EQE measurement system (Newport PV measurement, American).

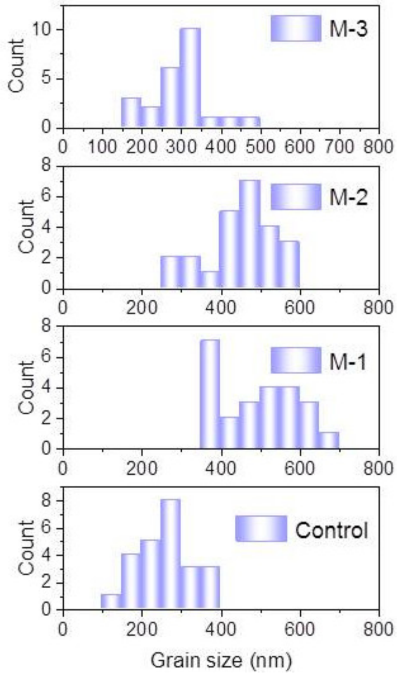

Figure S1. The grain sizes histogram for the perovskite film with different concentrations ABL additives.

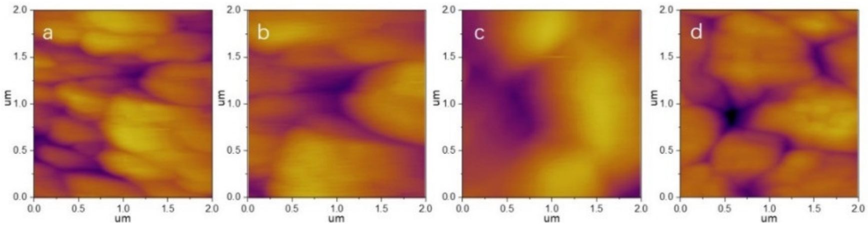

Figure S2. The AFM images of the perovskite films with different ABL additives: (a) control, 0 mg/mL, (b) M1, 0.1 mg/mL, (c) M2, 0.5 mg/mL, (d) M3, 1mg/mL.

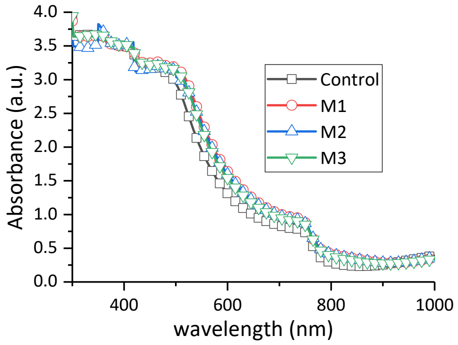

Figure S3. The absorption of the perovskite films with different concentrations ABL additives.

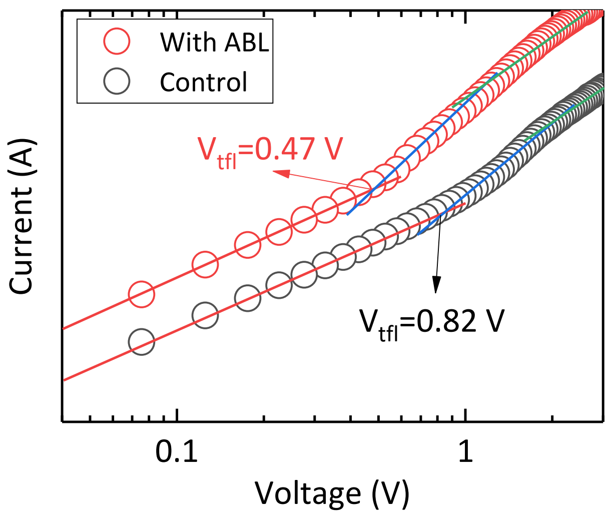

Figure S4. The *J–V* curves of electronic-dominated devices with a structure of glass/ITO/SnO_2/$MAPbI_3$ (with- and without- ABL)/PCBM/Ag. The trap density can be determined from V_{TFL} by referring to the relationship V_{TFL}=eN_tL^2/2εε$_0$, where the relative dielectric constant ε of $MAPbI_3$, the ε_0 is vacuum dielectric constant, and L is the thickness of the perovskite film.

.

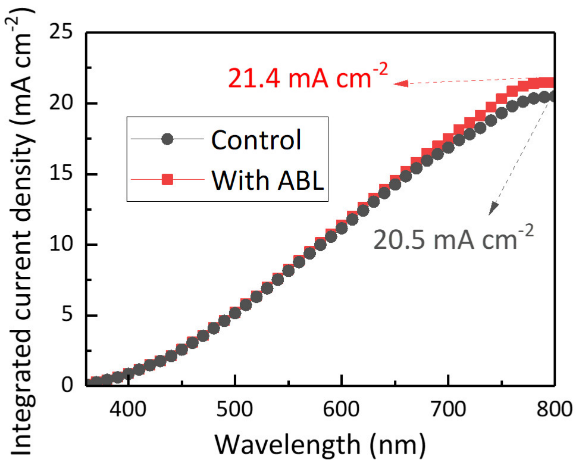

Figure S5. The integrated current density of the control and with ABL devices.

Table S1. TRPL lifetimes of the $MAPbI_3$ and $MAPbI_3$+ABL films.

Sample	τ_1 (ns)	τ_2 (ns)	τ_{avr} (ns)
$MAPbI_3$	49.3270	373.6547	152.6634
$MAPbI_3$+ABL	59.2856	1114.7028	468.9000

Table S2. The illuminated output paramenters of the PSCs with and without ABL.

Sample	V_{oc} (V)	J_{sc} (mA cm^{-2})	FF (%)	PCE (%)
Control R-S	1.09	21.66	77.52	18.30
Control F-S	1.08	21.32	75.37	17.35
With ABL R-S	1.13	23.08	80.04	20.87
With ABL F-S	1.12	22.99	79.06	20.36
